# Banhasasim-Tang Treatment Reduces the Severity of Esophageal Mucosal Ulcer on Chronic Acid Reflux Esophagitis in Rats

**DOI:** 10.1155/2017/7157212

**Published:** 2017-03-02

**Authors:** Mi-Rae Shin, Bu-Il Seo, Chang Gue Son, Seong-Soo Roh, Hyo-Jin An

**Affiliations:** ^1^Department of Pharmacology, College of Korean Medicine, Sangji University, Wonju-si, Gangwon-do 26339, Republic of Korea; ^2^Department of Herbology, College of Korean Medicine, Daegu Haany University, 136 Shinchendong-ro, Suseong-gu, Daegu 42158, Republic of Korea; ^3^Liver and Immunology Research Center, Daejeon Oriental Hospital, Oriental Medical College, Daejeon University, 176-9 Daeheung-ro, Jung-gu, Daejeon 34929, Republic of Korea

## Abstract

The present study was conducted to evaluate both antioxidant and anti-inflammatory activity of Banhasasim-tang (BHSST) on chronic acid reflux esophagitis (CRE) model. Rat CRE model was established operatively and then treated with BHSST (1 g/kg body weight per day) for 15 days Esophageal pathological changes were analyzed using macroscopic examination and hematoxylin/eosin staining. The antioxidant and inflammatory protein levels were determined using Western blotting. The administration of BHSST significantly reduced both the overexpression of serum reactive oxygen species (ROS) and an excessive formation of thiobarbituric acid-reactive substances (TBARS) in esophagus tissue. Thus, the severity of esophageal ulcer was lower in BHSST treated rats than control rats on the gross and histological evaluation. Nuclear factor-erythroid 2-related factor 2 (Nrf2) led to the upregulation of antioxidant enzyme including SOD, GPx-1/2, and HO-1 by binding to antioxidant response element (ARE). Moreover, BHSST administration markedly reduced the expression of inflammatory proteins through mitogen-activated protein kinase- (MAPK-) related signaling pathways and decreased significantly the protein expressions of inflammatory mediators and cytokines by inhibition of nuclear factor-kappa B (NF-*κ*B) activation. Taken together, these results support the fact that BHSST administration can suppress the development of esophageal mucosal ulcer* via* regulating inflammation through the activation of the antioxidant pathway.

## 1. Introduction

Gastroesophageal reflux disease (GERD) was defined as a condition that develops when the reflux of gastric contents into the esophagus causes troublesome symptoms such as acid regurgitation, heartburn, and dysphagia [1, 2]. Proton pump inhibitors (PPIs) for acid suppression are the mainstay therapeutic strategy for GERD and irreplaceable drugs in the management of acid-related disorders [3]. Despite their excellent efficacy, such agents have adverse effects associated with a long-term inappropriate use; namely, about one-third of patients with suspected GERD caused by weakly acidic reflux and duodenogastrooesophageal reflux experienced failure of PPIs [4–6]. Accordingly, the recent researches are focused on a new and rational approach about the effective and safe replacement therapy.

The cellular biochemical process for maintenance of homeostasis is regulated by redox balance. The redox imbalance may result in excessive production of reactive oxygen species (ROS) which may lead to oxidative stress. Redox imbalance/oxidative stress deteriorate when there is augmented production and inefficient scavenging of ROS [7, 8]. A continuous reflux of gastric contents causes inflammation, ulceration, and destruction of the normal squamous epithelium of esophagus. The damaged squamous epithelium of the esophagus is replaced with an intestine-like columnar epithelium which has strong resistance about acid attack and it is called Barrett's esophagus (BE). BE is commonly noted in humans with chronic reflux esophagitis and increases a risk for development of adenocarcinoma of the esophagus and gastric cardia [9, 10]. Accordingly, the suppression of the gastroesophageal reflux is the most important in treatment of GERD.

Banhasasim-tang (BHSST; Hange-shashin-to in Japanese Traditional Kampo Medicine; Banxia-xiexin-tang in Traditional Chinese Medicine) has been used as an herbal prescription to improve dyspepsia, gastric ulcerative disorders, laryngopharyngitis, colitis, and diarrhea [11–14]. This formula is composed of Pinelliae Rhizoma, Scutellariae Radix, Zingiberis Rhizoma Siccus, Ginseng Radix Alba, Glycyrrhizae Radix, Zizyphi Fructus, and Coptidis Rhizoma (Table 1). BHSST described in Shang-Han Lun (Treatise on Cold Damage and Miscellaneous Diseases) written by Zhang Zhong-jing (150–219 A.D.) is widely accepted by Chinese herbal doctors and has been applied for treating the symptom associated to GERD in Korea [15, 16]. Despite previous various reports related to the improvement of GERD, the protective mechanisms of BHSST treatment in esophageal ulcer by chronic reflux are not fully understood. Therefore, we investigated the effects of BHSST on rats with chronic acid reflux esophagitis (CRE) to examine its ameliorating effect against oxidative stress-related inflammation.

## 2. Materials and Methods

### 2.1. Materials

The protease inhibitor mixture solution and ethylenediaminetetraacetic acid (EDTA) were purchased from Wako Pure Chemical Industries, Ltd. (Osaka, Japan). Phenylmethylsulfonyl fluoride (PMSF) was purchased from Sigma-Aldrich (St. Louis, MO, USA). 2′,7′-Dichlorofluorescein diacetate (DCF-DA) was obtained from Molecular Probes (Eugene, OR, USA). The pierce bicinchoninic acid (BCA) protein assay kit was obtained from Thermo Fisher Scientific (Waltham, MA, USA). ECL Western Blotting Detection Reagents and pure nitrocellulose membranes were supplied by GE Healthcare (Chicago, IL, USA). Rabbit polyclonal antibodies against nuclear factor-kappa B p65 (NF-*κ*Bp65; 1 : 1,000, SC-372), nuclear factor-erythroid 2-related factor 2 (Nrf2; 1 : 1,000, SC-7228), heme oxygenase-1 (HO-1; 1 : 1,000, SC-10789), superoxide dismutase (SOD; 1 : 1,000, SC-11407), glutathione peroxidase-1/2 (GPx-1/2; 1 : 1,000, SC-30147); goat polyclonal antibodies against Kelch-like ECH-associated protein 1 (Keap1; 1 : 1000, SC-15246), tumor necrosis factor-*α* (TNF-*α*; 1 : 1,000, SC-1351), and interleukin-6 (IL-6; 1 : 1,000, SC-1266) and mouse monoclonal antibodies against phosphor-c-Jun NH_2_-terminal kinase (p-JNK; 1 : 1000, SC-6254), phosphor-extracellular signal-regulated kinase 1/2 (p-ERK1/2; 1 : 1000, SC-7383), phosphor-p38 (p-p38; 1 : 1000, SC-7973), cyclooxygenase-2 (COX-2; 1 : 1,000, SC-19999), inducible nitric oxide synthase (iNOS, 1 : 1,000, SC-7271), histone (1 : 1,000, SC-8030), and *β*-actin (1 : 1,000, SC-4778) were purchased from Santa Cruz Biotechnology, Inc. (Santa Cruz, CA, USA). Mouse monoclonal antibody against activator protein-1 (AP-1) subunit c-Jun (1 : 1000, #2315) was obtained from Cell Signaling Technology, Inc. (Cell Signaling, MA, USA). Rabbit anti-goat (1 : 3,000, SC-2774), goat anti-rabbit (1 : 3,000, SC-2004), and goat anti-mouse (1 : 3,000, SC-2005) immunoglobulin G (IgG) horseradish peroxidase- (HRP-) conjugated secondary antibodies were acquired from Santa Cruz Biotechnology, Inc. (Santa Cruz, CA, USA). All other chemicals and reagents were purchased from Sigma-Aldrich (St. Louis, MO, USA).

### 2.2. Test Material

Light brown granules of Banhasasim-tang (Hankook Shinyak Corp., Nonsan-si, Chungcheongnam-do, Republic of Korea) produced according to Korean Good Manufacturing Practice (GMP) were permitted and regulated by the Korean Food & Drug Administration (KFDA; Seoul, Republic of Korea). BHSST was dissolved in distilled water. The Banhasasimtang Ex. Granule is included as 1.5 g in Banhasasim-tang (1 package; 3.5 g) used in this study and illustrated in Table 1.

### 2.3. Experimental Animals and Treatment

Animal experiments were carried out according to the “Guidelines for Animal Experimentation” approved by the Ethics Committee of the Daegu Haany University on 20/10/2016 with certificate number DHU2016-81. Five-week-old male Sprague-Dawley rats (B.W. 150–155 g) were purchased from Nara Biotec Co. (Pyeongtaek, Republic of Korea). Rats were maintained under a 12 h light/dark cycle, housed at controlled temperature (24 ± 2°C) and humidity (about 60%), and kept in raised mesh-bottom cages to prevent coprophagy. After adaptation (1 week), the rats were fasted for 18 h prior to surgical procedures and kept in raised mesh-bottom cages to prevent coprophagy. And then rats were anaesthetized with an injection of Zoletil at 0.75 mg/kg (Virbac SA, France). The CRE model (chronic acid reflux esophagitis model) was developed by following the methods proposed by Omura et al. [17]. A midline laparotomy was performed to expose the stomach and the transitional region (i.e., limiting ridge) between the fundus and the glandular portion of the stomach was ligated with 2–0 silk thread in order to restrict the compliance of the stomach, which led to the reflux of gastric contents into the esophagus. Additionally, a latex ring (2 mm in thickness; ID, 4 mm, made from 18-Fr Nelaton catheter) was placed around the pyloric sphincter so as to restrict the emptying of gastric contents. Rats were injected with gentamicin sulfate (antibiotic, subcutaneous injection) and dexamethasone (anti-inflammatory agent, subcutaneous injection) for 3 days to prevent infection. After surgery, the rats fasted for a further 48 h but water was provided 24 h after surgery. All animals had an operation adjustment for 7 days after surgery. Body weight was recorded during 22 days from surgery day (an operation adjustment period; 7 days + drug treatment period; 15 days) and food intake was recorded during 15 days (drug treatment period). At 22 days after surgery, rats were sacrificed and the esophageal tissues were obtained for further processing and analysis. Rats were divided into three groups. The normal and chronic acid reflux esophagitis control groups were given water, while the drug group was orally administered with Banhasasim-tang at a dose of 1 g/kg body weight daily using a stomach tube for 15 consecutive days (*n* = 7 in each group). The entire esophagus was removed immediately and examined for gross mucosal injury. The esophageal tissue was immediately frozen in liquid nitrogen and blood samples were collected by vena cava puncture from anesthetized rats. Subsequently, the esophagus and serum were kept at −80°C until analysis.

### 2.4. Esophageal Ulcer Ratio

The rat esophagus was cut with scissors in a longitudinal direction from the gastroesophageal junction to the pharynx after sacrifice. The inner mucous was washed away with 0.9% NaCl and the remaining tissue was laid out on paper. Thereafter, the dissected esophagus was photographed with an optical digital camera (Sony, Tokyo, Japan) and analyzed using the i-solution lite software program. The gross mucosal ulcer ratio was calculated as follows: The gross mucosal ulcer ratio (%) = [width of area with esophageal mucosal ulcer (mm^2^)/width of total area of esophagus (mm^2^)] × 100.

### 2.5. ROS and TBARS Levels Measurements

Serum ROS level was measured employing the method of Ali et al. [18]. Esophageal tissues were homogenized on ice with 1 mM EDTA-50 mM sodium phosphate buffer (pH 7.4), and then 25 mM DCF-DA was added to homogenates. After incubation for 30 min, the changes in fluorescence values were determined at an excitation wavelength of 486 nm and emission wavelength of 530 nm. The 2-thiobarbituric acid-reactive substance (TBARS) level was estimated according to the method of Mihara and Uchiyama [19].

### 2.6. Preparation of Cytosol and Nuclear Fractions

Protein extraction was performed according to the method of Komatsu with minor modifications [20]. Esophageal tissues for cytosol fraction were homogenized with ice-cold lysis buffer A (250 mL) containing 10 mM HEPES (pH 7.8), 10 mM KCl, 2 mM MgCl_2_, 1 mM DTT, 0.1 mM EDTA, 0.1 mM PMSF, and 1,250 *μ*L protease inhibitor mixture solution. The homogenate was incubated at 4°C for 20 min. And then 10% NP-40 was added and mixed well. After centrifugation (13,400 ×g for 2 min at 4°C) using Eppendorf 5415R (Hamburg, Germany), the supernatant liquid (cytosol fraction) was separated in new e-tube. The left pellets were washed twice by buffer A and the supernatant was discarded. Next, the pellets were suspended with lysis buffer C (20 mL) containing 50 mM HEPES (pH 7.8), 50 mM KCl, 300 mM NaCl, 1 mM DTT, 0.1 mM EDTA, 0.1 mM PMSF, 1% (v/v) glycerol, and 100 *μ*L protease inhibitor mixture solution suspended and incubated at 4°C for 30 min. After centrifugation (13,400 ×g for 10 min at 4°C), the nuclear fraction was prepared to collect the supernatant. Both cytosol and nuclear fractions were kept at −80°C before the analysis.

### 2.7. Immunoblotting Analyses

For the estimation of Nrf2, NF-*κ*Bp65, and histone, 13.6 *μ*g of protein from each nuclear fraction was electrophoresed through 8–10% sodium dodecylsulfate polyacrylamide gel (SDS-PAGE). Separated proteins were transferred to a nitrocellulose membrane, blocked with 5% (w/v) skim milk solution for 1 h, and then incubated with primary antibodies (Nrf2, NF-*κ*Bp65, and histone) overnight at 4°C. After the blots were washed, they were incubated with anti-rabbit or anti-mouse IgG HRP-conjugated secondary antibody for 1 h at room temperature. In addition, 8 *μ*g protein of each cytosol fraction of Keap1, SOD, GPx-1/2, HO-1, COX-2, iNOS, TNF-*α*, IL-6, and *β*-actin was electrophoresed through 8–15% SDS-PAGE. Each antigen-antibody complex was visualized using ECL Western Blotting Detection Reagents and detected by chemiluminescence with Sensi-Q 2000 Chemidoc (Lugen Sci Co., Ltd., Gyeonggi-do, Republic of Korea). Band densities were measured using ATTO Densitograph Software (ATTO Corporation, Tokyo, Japan) and quantified as the ratio to histone or *β*-actin. The protein levels of the groups are expressed relative to those of the normal rat (represented as 1).

### 2.8. Statistical Analysis

The data are expressed as the mean ± SEM. Significance was assessed by one-way analysis of variance (ANOVA) followed by Dunnett's multiple comparison test using SPSS version 22.0 software (SPSS Inc., Chicago, IL, USA). Values of *P* < 0.05 were considered significant.

## 3. Results and Discussions

**Figure 1:**
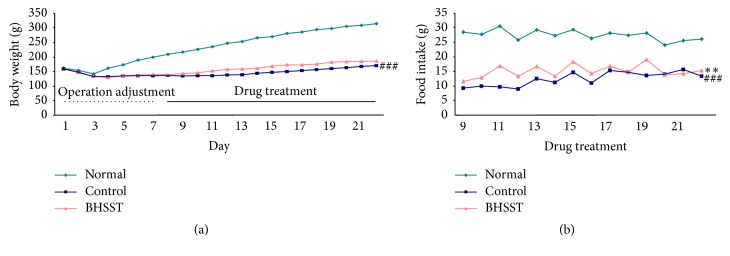
Body weight changes in the whole experimental periods (a) and food intake changes in the drug treatment periods (b) in chronic acid reflux esophagitis rats. Normal, normal rats; Control, chronic acid reflux esophagitis rats; BHSST, BHSST 1 g/kg body weight/day-treated chronic acid reflux esophagitis rats. Data are mean ± SEM (*n* = 7). Significance: ^###^*P* < 0.001 versus normal rats and ^*∗∗*^*P* < 0.01 versus control rats.

Traditional herbal formulas have been widely used to prevent and treat various inflammatory disorders in East Asia, including Korea, China, and Japan. BHSST among these formulas consists of seven herbs, Pinelliae Rhizoma, Scutellariae Radix, Zingiberis Rhizoma Siccus, Ginseng Radix Alba, Glycyrrhizae Radix, Zizyphi Fructus, and Coptidis Rhizoma in 5 : 3 : 3 : 3 : 3 : 3 : 1 proportions. The previous study reported that the major thirteen marker components of BHSST using an ultra-performance liquid chromatography coupled to electrospray ionization tandem mass spectrometry are homogentisic acid (Pinelliae Rhizoma), 3,4-dihydroxybenzaldehyde (Pinelliae Rhizoma), spinosin (Zizyphi Fructus), liquiritin (Glycyrrhizae Radix), baicalin (Scutellariae Radix), ginsenoside Rg1 (Ginseng Radix Alba), liquiritigenin (Glycyrrhizae Radix), wogonoside (Scutellariae Radix), ginsenoside Rb1 (Ginseng Radix Alba), baicalein (Scutellariae Radix), glycyrrhizin (Glycyrrhizae Radix), wogonin (Scutellariae Radix), and 6-gingerol (Zingiberis Rhizoma Siccus) [21]. Particularly, the highest content is baicalin and then wogonoside, glycyrrhizin, liquiritin, and 6-gingerol. Moreover, the existing studies demonstrated that Pinelliae Rhizoma [22], Scutellariae Radix [23], Zingiberis Rhizoma Siccus [24], Ginseng Radix Alba [25], Glycyrrhizae Radix [26], Zizyphi Fructus [27], and Coptidis Rhizoma [28] exert the gastroprotective effects through inhibition of inflammatory proteins. Based on previous studies, we predicted that BHSST, containing these bioactive herbs as constituents, would enhance ameliorating effects on esophageal ulcer induced chronic acid reflux esophagitis. Therefore, the present study was conducted using the same chronic acid reflux esophagitis model used in the previous experimental [17]. First of all, body weight gain during the experimental periods and food intake during drug treatment periods are confirmed. As shown in Figure 1(a), normal rats and chronic acid reflux esophagitis (CRE) model induced rats are similar to body weight in starting-point. Body weight decreased 3 days in a row after surgery whereas it increased gradually from 4 days. Based on the whole experimental periods, control rats significantly suffered weight loss (^###^*P* < 0.001) compared with normal rats due to the low food intake. However, the significant increase of food intake during drug treatment periods by BHSST treatment (^*∗∗*^*P* < 0.01) leads to the body weight rise (without a significance) (Figure 1(b)). The results suggested that changes in the food intake and body weight were caused by surgery assuming a comparative similar aspect [29].

**Figure 2:**
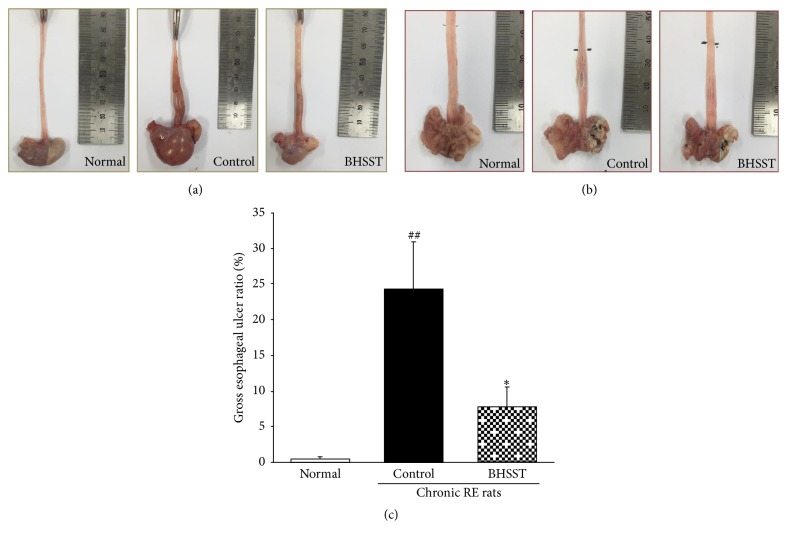
Gross esophagus (a), the opened gross esophageal ulcer (b), and esophageal mucosal ulcer ratio (c) in chronic acid reflux esophagitis rats. Normal, normal rats; Control, chronic acid reflux esophagitis rats; BHSST, BHSST 1 g/kg body weight/day-treated chronic acid reflux esophagitis rats. Data are mean ± SEM (*n* = 7). Significance: ^##^*P* < 0.01 versus normal rats and ^*∗*^*P* < 0.05 versus control rats.

**Figure 3:**
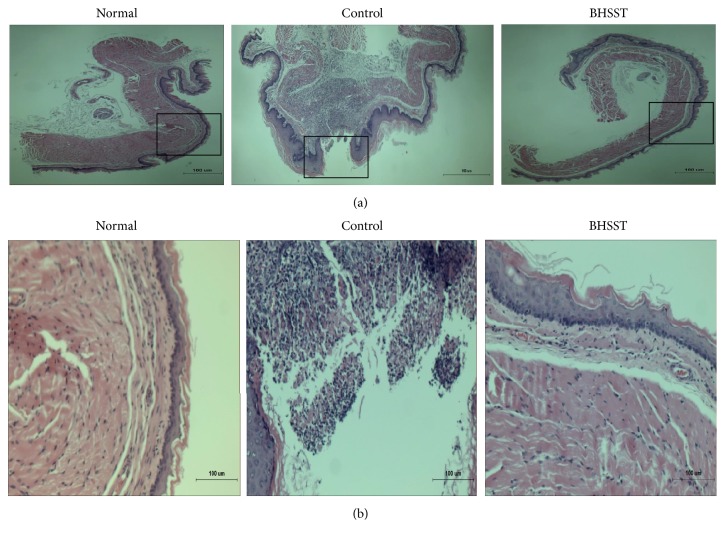
H/E staining of esophageal ulcer in chronic acid reflux esophagitis rats. (a) Magnification ×40 and (b) magnification ×200. Normal, normal rats; Control, chronic acid reflux esophagitis rats; BHSST, BHSST 1 g/kg body weight/day-treated chronic acid reflux esophagitis rats.

Gross morphological changes such as mucosal swelling and esophageal ulcer ratio as shown in Figure 2 associated with the metaplastic process of mucosal epithelial cell were observed in chronic reflux esophagitis rats instead of hyperemia and multiple erosions showed in acute reflux esophagitis [30]. Herein, the different severity of esophageal ulceration was seen in rats with CRE (Figure 2(c)), while no visible esophageal mucosa lesions were observed in normal rats. These tissue injuries were located in the middle or distal esophagus. Moreover, the normal esophagus exhibited a thin epithelial layer with squamous cells and few inflammatory cells in the submucosal layer, while the CRE esophagus exhibited basal layer thickening, inflammatory cells infiltration, and the desquamated epithelial cells (Figure 3) [31]. However, the CRE rats treated with BHSST had less damage than the CRE control rats. These results were consistent with the histomorphological staining results.

**Figure 4:**
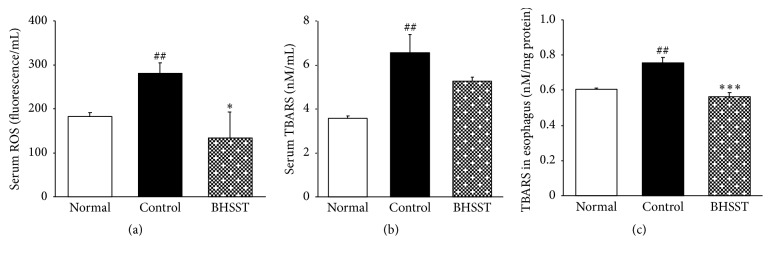
ROS and TBARS levels measurements. (a) Serum ROS, (b) serum TBARS, and (c) TBARS of esophageal tissue. Normal, normal rats; Control, chronic acid reflux esophagitis rats; BHSST, BHSST 1 g/kg body weight/day-treated chronic acid reflux esophagitis rats. Data are mean ± SEM (*n* = 6). Significance: ^##^*P* < 0.01 versus normal rats and ^*∗*^*P* < 0.05, ^*∗∗∗*^*P* < 0.001 versus control rats.

Several herbal therapies have been proposed for the treatment of GERD [32]; however, the role of BHSST against CRE still lacks proved experiments. In the current study, we clearly demonstrated that supplementation of BHSST significantly ameliorated chronic acid reflux esophagitis- (CRE-) induced esophageal ulcer. The severity of an esophageal ulcer has been correlated with the overproduction of free radicals. Oxidative stress (OS) is caused when production of reactive oxidative species (ROS) exceeds the potential of cellular antioxidant defenses to detoxify these toxicants [33]. And then, esophageal ulcer by the continuous and chronic reflux progresses gradually and can deteriorate until esophageal stricture or Barret's esophagus. Ultimately, it may contribute to the development of esophageal cancer [34]. OS can be determined by measuring the levels of malondialdehyde (MDA), which is a lipid peroxide generated by the reaction among oxygen free radicals [35]. MDA is reactive marker of membrane damage and forms a colour complex with thiobarbituric acid (TBA) which can be measured spectrophotometrically. Herein, we measured the content of MDA using the method of TBARS assay. In present study, the levels of serum ROS and esophageal TBARS were markedly higher than those of normal rats, whereas the elevated levels of serum ROS were significantly decreased nearly to the levels of normal rats. On the other hand, serum TBARS showed a tendency to reduction (without significance) (Figure 4).

**Figure 5:**
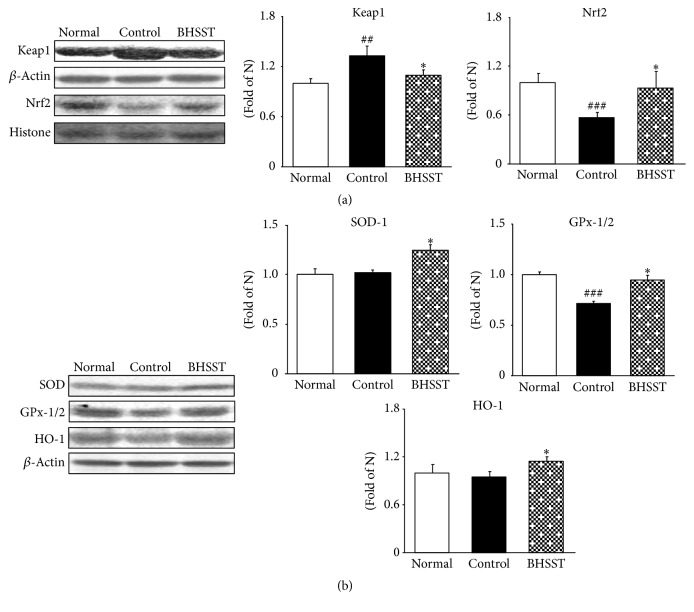
Effect of BHSST on antioxidant proteins in chronic acid reflux esophagitis rats. (a) Nrf2 and Keap1 protein expressions and (b) SOD, GPx-1/2, and HO-1 protein expressions. Normal, normal rats; Control, chronic acid reflux esophagitis rats; BHSST, BHSST 1 g/kg body weight/day-treated chronic acid reflux esophagitis rats. Data are mean ± SEM (*n* = 6). Significance: ^##^*P* < 0.01, ^###^*P* < 0.001 versus normal rats and ^*∗*^*P* < 0.05 versus control rats.

A major cellular defense mechanism, which is sensitive to oxidative stress, is the nuclear factor E2-related factor 2/Kelch-like ECH-associated protein 1/antioxidant response elements (Nrf2-Keap1-ARE) pathway and activates as adaptive response to protect cells from injury [36, 37]. In normal cells, Nrf2 is sequestered by the Keap1 to form Nrf2-Keap1 complex. However, during oxidative stress Nrf2 dissociates from Keap1, translocates into the nucleus, and binds to ARE, promoting the transcription of the target gene [38]. As shown in Figure 5(a), the Nrf2 protein expression was decreased in control rats compared with normal rats whereas BHSST treatment significantly upregulated Nrf2 expression. Moreover, Keap1 level of control rats was higher than that of the normal rats but it significantly decreased by BHSST administration. Particularly, ARE characterizes its unique responsiveness to oxidative stress. Thus, change of the cellular redox status due to ROS overexpression and/or a reduced antioxidant capacity appears to be an important signal for triggering the transcriptional response [39]. Activation of Nrf2 signaling induces the transcriptional regulation of ARE-dependent expression of antioxidant defense enzymes (such as SOD, GPx, and HO-1). In our results, chronic acid reflux esophagitis rats showed decreased expressions of Nrf2, SOD, GPx-1/2, and HO-1 in esophageal tissues compared with normal rats; however, BHSST administration effectively upregulated Nrf2 and alleviated oxidative stress. Besides, antioxidant enzyme including SOD, GPx-1/2, and HO-1 significantly increased compared with control rats (Figure 5(b)).

A low level of OS induces Nrf2, a transcription factor related to the transactivation of gene coding for antioxidant enzymes. An intermediate amount of OS triggers an inflammatory response through the activation of NF-*κ*B whereas a high level of OS results in apoptosis or necrosis [40]. Thereby, Nrf2 and NF-*κ*B pathways can be functionally antagonistic to control the transcription or function of their downstream targets. Namely, Nrf2 encodes for antioxidant and general cytoprotection genes, while NF-*κ*B regulates the expression of proinflammatory genes. So, the activation of Nrf2-mediated antioxidant signaling attenuates NF-*κ*B-mediated inflammatory response. As the result, activation of NF-*κ*B pathway impairs esophageal barrier function and activation of Nrf2 pathway may play a protective role.

**Figure 6:**
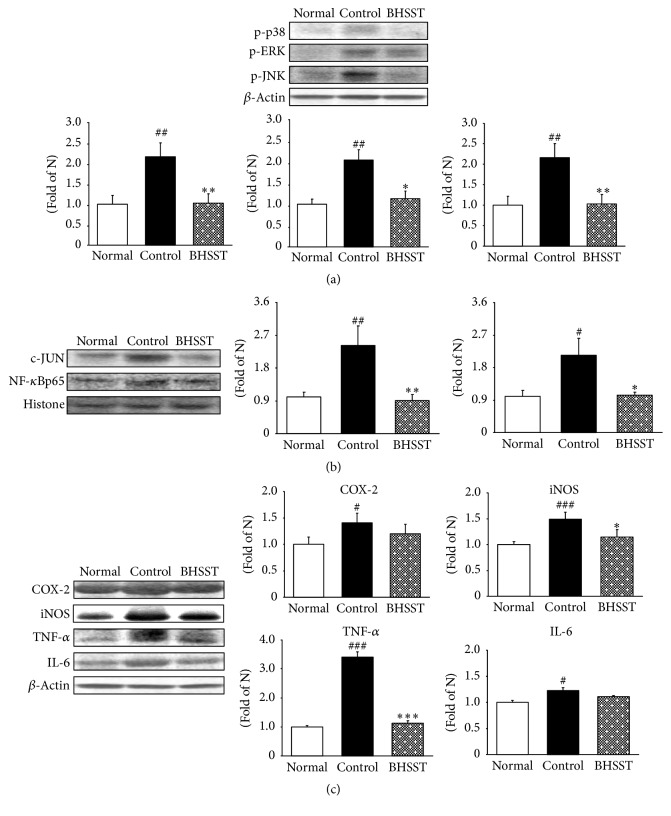
Effect of BHSST on proinflammatory proteins in chronic acid reflux esophagitis rats. (a) p-p38, p-ERK1/2, and p-JNK protein expressions; (b) c-JUN and NF-*κ*Bp65 protein expressions; (c) COX-2, iNOS, TNF-*α*, and IL-6 protein expressions. Normal, normal rats; Control, chronic acid reflux esophagitis rats; BHSST, BHSST 1 g/kg body weight/day-treated chronic acid reflux esophagitis rats. Data are mean ± SEM (*n* = 6). Significance: ^#^*P* < 0.05, ^##^*P* < 0.01, and ^###^*P* < 0.001 versus normal rats and ^*∗*^*P* < 0.05, ^*∗∗*^*P* < 0.01, and ^*∗∗∗*^*P* < 0.001 versus control rats.

Mitogen-activated protein kinase (MAPK) signal transduction pathways are among the most widespread mechanisms that can be activated by a variety of stimuli including oxidative stress. The 3 principal MAPK components are the extracellular signal-regulated kinase (ERK), c-Jun N-terminal kinase (JNK), and p38 MAPK [41]. MAPK pathways activated by acid exposure found in the metaplastic esophageal mucosa of patients with Barrett's esophagus [42]. Moreover, ERK1/2 and p38 MAPK can intensify the expression of these inflammatory factors such as COX-2 and prostaglandin (PGE) 2 [43]. JNK, which is activated by environmental stress, phosphorylates and regulates the activity of transcription factors including c-Jun. Moreover, JNK is also activated by proinflammatory cytokines, such as TNF and IL-1 [44]. In our study, MAPK-related protein expressions were significantly augmented in the esophagus of control rats compared to the normal rats, but the oral administration of BHSST markedly lowers the expressions of p-p38, p-ERK1/2, and p-JNK as shown in Figure 6(a).

One of the well-studied transcription factors downstream MAPKs signaling is the nuclear factor NF-*κ*B. The phosphorylation of p38 and ERK1/2 MAPK leads to NF-*κ*B translocation and NF-*κ*B promotes the transcription of target genes such as TNF-*α* and IL-6 and regulates the expression of inducible enzymes such as COX-2 and iNOS [45]. The protein expressions of c-Jun and NF-*κ*B in control rats were significantly induced to a greater extent compared with normal rats. The administration of BHSST led to significant downregulation of these two transcription protein expressions (Figure 6(b)). The results of the present study show that BHSST promotes the suppression of NF-*κ*B activation in esophageal tissue. As the result, upregulation of NF-*κ*B-related inflammatory mediators (iNOS) significantly decreased. In addition, the elevated TNF-*α* level was significantly lowered by the administration of BHSST; otherwise COX-2 and IL-6 showed a tendency to decrease (without significance) in the esophagus (Figure 6(c)).

**Figure 7:**
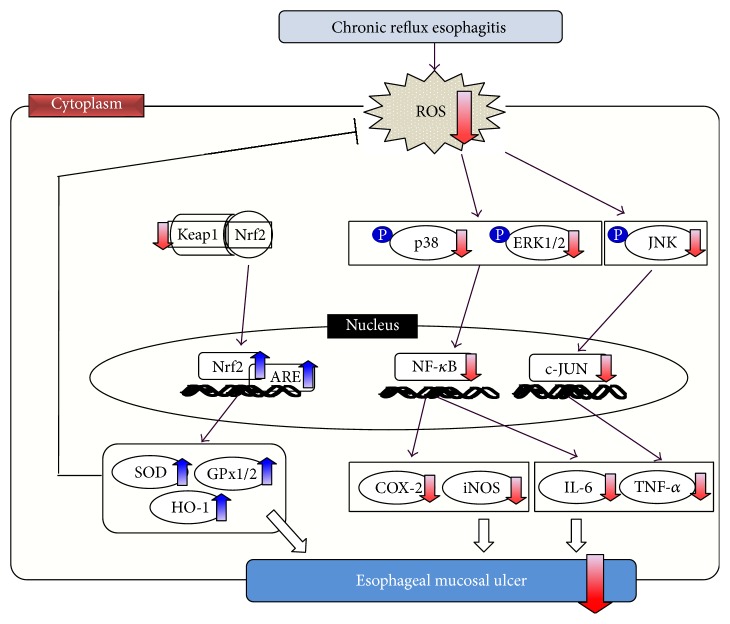
Possible mechanism of BHSST in the esophagus of chronic acid reflux-induced esophageal ulcer rats.

A recent study showed that ROS are one of the most important factors in the pathogenesis of esophageal mucosal injury mediated by oxidative stress in an experimental model of reflux esophagitis. In the present study, the administration of BHSST reduced the oxidative stress via Nrf2/Keap1/ARE pathway. Furthermore, the anti-inflammatory effect of BHSST suggested that the blocking MAPK such as p38, ERK1/2, and JNK signaling pathways and NF-*κ*B inactivation led to an inhibition of the release of proinflammatory cytokines and mediators. That is, BHSST ameliorated esophageal ulcer caused by experimental reflux esophagitis in rats, as shown in Figure 7. Nevertheless, the action mechanism of BHSST is still ambiguous and further profound researches are required.

## 4. Conclusions

In conclusion, BHSST was associated with downregulation of proinflammatory proteins and appeared to contribute to the antioxidant defense. Based on these, BHSST treatment showed the amelioration of esophageal mucosal ulcer which is one of chronic GERD symptoms. Therefore, these data may provide a scientific basis for BHSST to expand the indication in the management of GERD field.

## Figures and Tables

**Table 1:** Composition of Banhasasim-tang (BHSST) used in this study.

Herbs	Amounts (g)
Pinelliae Rhizoma	1.67
Scutellariae Radix	1.00
Zingiberis Rhizoma Siccus	0.83
Ginseng Radix	1.00
Glycyrrhizae Radix	1.00
Jujubae Fructus	1.00
Coptidis Rhizoma	0.33

Banhasasimtang Ex. Granule (1.5 g) is contained in 1 pack (3.5 g).

Banhasasimtang Ex. Granule was purchased from Hankook Shinyak Corp. (Nonsan-si, Chungcheongnam-do, Republic of Korea).
